# Plant Functional Genomics Based on High‐Throughput CRISPR Library Knockout Screening: A Perspective

**DOI:** 10.1002/ggn2.202300203

**Published:** 2023-11-27

**Authors:** Jianjie He, Can Zeng, Maoteng Li

**Affiliations:** ^1^ Department of Biotechnology College of Life Science and Technology Huazhong University of Science and Technology Wuhan 430074 China; ^2^ Key Laboratory of Molecular Biophysics of the Ministry of Education Wuhan 430074 China

**Keywords:** CRISPR library, functional genomics, high throughput, knockout, mutant collection

## Abstract

Plant biology studies in the post‐genome era have been focused on annotating genome sequences’ functions. The established plant mutant collections have greatly accelerated functional genomics research in the past few decades. However, most plant genome sequences' roles and the underlying regulatory networks remain substantially unknown. Clustered, regularly interspaced short palindromic repeat (CRISPR)‐associated systems are robust, versatile tools for manipulating plant genomes with various targeted DNA perturbations, providing an excellent opportunity for high‐throughput interrogation of DNA elements’ roles. This study compares methods frequently used for plant functional genomics and then discusses different DNA multi‐targeted strategies to overcome gene redundancy using the CRISPR‐Cas9 system. Next, this work summarizes recent reports using CRISPR libraries for high‐throughput gene knockout and function discoveries in plants. Finally, this work envisions the future perspective of optimizing and leveraging CRISPR library screening in plant genomes' other uncharacterized DNA sequences.

## Graphical abstract

This study compares methods frequently used for plant functional genomics and then discusses different DNA multi‐targeted strategies using clustered, regularly interspaced short palindromic repeat (CRISPR)‐Cas9. Next, this work summarizes recent reports using CRISPR libraries for high‐throughput gene knockout and function discoveries in plants. Finally, this work envisions the future perspective of optimizing and leveraging CRISPR library screening in plant genomes' other largely unknown DNA sequences.

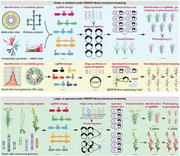

## 1 Introduction

Since the release of *Arabidopsis thaliana*’s genome sequence,^[^ ^1^, ^2^, ^3^, ^4^, ^5^ ^]^ more than 3150 plant genomes have been sequenced and assembled during the past two decades.^[^ ^6^ ^]^ Most recently, implicated by the first complete sequence of human genomes,^[^ ^7^, ^8^, ^9^ ^]^ the telomere‐to‐telomere gap‐free assembly of plant genomes has quickly exploded,^[^ ^10^, ^11^, ^12^, ^13^, ^14^, ^15^ ^]^ accelerating the development of novel bioinformatics platforms for comparative genomics.^[^ ^16^ ^]^ These valuable complete plant genome sequences and sophisticated bioinformatics tools will revolutionize crop breeding.^[^ ^17^ ^]^ However, compared to the fast advancement of genomics, functional annotation of these abundant genome resources lags far behind. It is mainly caused by limited tools for DNA perturbation and the laborious experimental process, such as generating loss‐of‐function mutants. Nonetheless, rapidly breeding crops with beneficial superior traits by manipulating their genomes, instead of selection by natural mutation, is essential to cope with the ever‐changing climate and feed the ever‐growing population globe.

Creating mutant collections by random or targeted mutagenesis is a practical approach to plant functional genomics and crop breeding. Many plant mutant libraries have been established over the past years, providing precious resources to the research community and breeding programs.^[^ ^18^, ^19^ ^]^ Recently, clustered, regularly interspaced short palindromic repeat (CRISPR)‐associated (Cas) nuclease system and its diversified tools have been leveraged to various targeted DNA perturbations across life kingdoms.^[^ ^20^ ^]^ CRISPR‐Cas9 system,^[^ ^21^ ^]^ among the other tools, such as adenine base editor,^[^ ^22^ ^]^ cytosine base editor,^[^ ^23^ ^]^ or prime editor,^[^ ^24^ ^]^ is the most frequently used technique in plant biology research and crop breeding.^[^ ^25^, ^26^, ^27^, ^28^, ^29^ ^]^ For example, it has been used for the domestication of wild plants^[^ ^30^, ^31^, ^32^ ^]^ and to generate clonal seeds.^[^ ^33^ ^]^ The essence that CRISPR‐Cas9‐based targeted editing only relies on the complex of a single‐guide RNA (sgRNA) and a Cas9 protein makes it feasible for high‐throughput CRISPR library screening. Thus far, this system has been used for directed evolution and generating knockout mutant collections in plants using pooled CRISPR libraries.^[^ ^34^, ^35^ ^]^

## 2 Methods for Plant Functional Genomics

Interrogation of gene functions requires many methods of genetic engineering.^[^ ^36^ ^]^ Below, we discuss the three frequently utilized strategies for plant functional genomics: gain‐of‐function technique, gene knockdown strategy, and loss‐of‐function approach. Then, we compare the strengths and shortcomings of these methods (**Table** **1**).

**Table 1:** A comparison of the methods used for plant functional genomics

Category^a)^	Method^b)^	Induced perturbation	Off‐target	Ease of large‐scale application	Causative gene(s) verification	Ability to disturb multigenes
GOF	CRISPRa	Targeted	Low	Yes	Easy	High
GOF	Activation tag	Random	None	Yes	Complicated	Null
GOF	FOX‐hunting	Targeted	None	Yes	Easy	Low
Knockdown	CRISPRi	Targeted	Low	Yes	Easy	High
Knockdown	VIGS	Targeted	Low	No	Easy	High
Knockdown	LhpRNA	Targeted	Low	Yes	Easy	High
LOF	EMS	Random	High	Yes	Difficult	Low
LOF	T‐DNA	Random	Low	Yes	Moderate	Low
LOF	TALEN/ZFN	Targeted	Low	No	Easy	High
LOF	CRISPRko	Targeted	Low	Yes	Easy	High

^a)^ Abbreviations: GOF, gain‐of‐function; LOF, loss‐of‐function;

^b)^ EMS and T‐DNA exemplify physicochemical treatment and biological insertion, respectively.

### 2.1 Gain‐of‐Function Strategy

The gain‐of‐function strategy refers to the methods by which the transcriptional product of the target gene is increased, thus enhancing its biological function. In this regard, this kind of genetic manipulation is almost always dominant or semi‐dominant. Thus far, two methods have been commonly utilized for large‐scale gain‐of‐function studies: activation tagging and FOX‐hunting system. Recently, the activation system based on CRISPR‐Cas (CRISPRa), which contains a sgRNA and a dead Cas protein (dCas) fused with transcriptional activators, such as VP64 and EDLL, has been leveraged for gene transcriptional activation in plants.^[^ ^37^, ^38^, ^39^, ^40^ ^]^ For example, Lowder et al. reported that the dCas9‐VP64 system increased the mRNA product of the *PAP1* gene to seven‐fold and the mRNA product of the *FIS2* gene to a maximum of 200‐fold in *A. thaliana*.^[^ ^39^ ^]^ Their further experiments showed that dCas9‐VP64 coupled with gRNA2.0, which recruited fused MS2‐VP64, could increase the mRNA of the *PAP1* gene to 30‐ to 45‐fold and the mRNA of the *FIS2* gene up to 1500‐fold.^[^ ^39^ ^]^ Nonetheless, their use for high‐throughput studies has yet to be reported.

The activation tagging technique typically leverages four copies of the engineered enhancer element to enhance the expression of the flanked genes of randomly inserted T‐DNA.^[^ ^41^, ^42^ ^]^ This technology often harnesses the *Ac*/*Ds* transposon or Cre‐Lox system to function effectively.^[^ ^43^ ^]^ The activation tagging strategy has been applied to generate many gain‐of‐function transgenic collections in plants such as *A. thaliana*,^[^ ^42^, ^44^ ^]^ rice,^[^ ^45^, ^46^ ^]^ and tomato.^[^ ^47^ ^]^ For instance, Weigel et al. have developed 49,000 *Arabidopsis* activation tagging lines to characterize various transformants showing different phenotypes, e.g., flowering time, disease resistance, and fruit morphology.^[^ ^42^ ^]^ This collection of activation tagging lines has resulted in many gene function discoveries. For instance, by screening 18,000 lines of this collection, Deng et al. identified the *AtMYB68*, a transcription factor in *A. thaliana*, as a critical player in heat and drought tolerance at yield‐determining reproductive stages.^[^ ^48^ ^]^ Another screening discovered a new gene, *AtPGX2*, which boosts hypocotyl elongation, leaf expansion, stem lignification, mechanical stiffening, and lodging in *A. thaliana*.^[^ ^49^ ^]^

In contrast to activating the endogenous genes by activation tagging, the FOX‐hunting (Full‐length cDNA Over‐eXpressing) system is associated with cloning the full‐length cDNAs to express them under constitutive promoters ectopically.^[^ ^50^ ^]^ This method has been used to create large gain‐of‐function collections in plants such as *A. thaliana* ^[^ ^50^, ^51^ ^]^ and rice.^[^ ^52^ ^]^ For instance, Nakamura et al. harnessed 13,980 independent full‐length cDNA clones to produce the FOX library, generating around 12,000 FOX‐rice lines.^[^ ^52^ ^]^ The authors characterized three dwarf rice transformants that overexpressed a novel gibberellin 2‐oxidase gene to elucidate the usefulness of this system. Another study transformed cDNA from *Brassica napus* seeds to *A. thaliana*, generating 4298 positive T_1_ transgenic plants.^[^ ^51^ ^]^ Leveraging this transgenic collection, the authors identified the *BnACBP1‐like* gene as an accelerator of the early leaf senescence in *B. napus*, and the overexpression of this gene accumulated jasmonic acid and oxylipins.^[^ ^51^ ^]^

### 2.2 Gene Knockdown Strategy

Gene knockdown refers to the expression of one or more genes being reduced through genetic manipulation. Gene knockdown (silencing) strategies used in plants consist of transcriptional gene silencing (TGS) and post‐transcriptional gene silencing (PTGS). Recently, CRISPR‐based TGS (CRISPRi) has been successfully achieved in plants.^[^ ^53^, ^54^ ^]^ CRISPRi silencer comprises a sgRNA and a dead Cas protein, e.g., dCpf1, fused with a repressor domain such as the plant SRDX,^[^ ^53^, ^54^ ^]^ which has shown the ability to operate plant transcriptome. For example, Tang et al. reported that the CRISPR‐dCpf1‐SRDX system could inhibit the expression of *miR159b*, a non‐coding RNA, to less than 10% of the *A. thaliana* wild type.^[^ ^54^ ^]^ Methods for PTGS in plants commonly include virus‐induced gene silencing (VIGS) and long hairpin RNA (lhpRNA) transgenes‐mediated RNAi. VIGS exploits the plant's intrinsic RNA‐mediated antiviral defense mechanism to target the viral genome upon the infection.^[^ ^55^ ^]^ Upon the infection, the engineered virus harboring a fragment of the host plant gene will lead to a homology‐based knockdown of the endogenous gene.^[^ ^56^ ^]^ For example, Zhou et al. used the tobacco rattle virus (TRV) to infect the Chinese narcissus.^[^ ^57^ ^]^ Their results showed that pTRV2‐*NtPDS* and pTRV2‐*NtMYB3* systems could efficiently silence the *NtPDS* and *NtMYB3* genes by less than 25% expression of the wild type, respectively.^[^ ^57^ ^]^ Another research reported using foxtail mosaic virus to establish the VIGS in switchgrass by testing the silencing efficiency of *ChlD*, *ChlI*, and *PDS* genes.^[^ ^58^ ^]^ The authors showed that silencing efficiency was more robust in leaves (≈63–94%) than in roots (≈48–78%).^[^ ^58^ ^]^ The other frequently adopted method for PTGS is mediated by lhpRNA transgenes, processed by plants’ endogenous machinery, generating small interfering RNAs (siRNAs) 20–24 nt in length for effective gene silencing.^[^ ^59^ ^]^ This strategy has been leveraged to create a genome‐wide RNAi rice library comprising over 6000 transgenic lines.^[^ ^59^ ^]^ Using this collection, the authors shed light on the functions of several genes in rice. For example, knockdown of the *Os08g0558600* gene could lead to a reduced tiller number phenotype with no heading.^[^ ^59^ ^]^ Nonetheless, it has yet to be a report regarding the large‐scale gene knockdown research using CRISPRi and VIGS.

### 2.3 Loss‐of‐Function Strategy

The loss‐of‐function strategy includes random and targeted mutagenesis. Typically used random mutagenesis approaches are physical or chemical treatments‐mediated point mutation and biological insertional mutation.^[^ ^60^ ^]^ Physical pollination in plants typically uses gamma or fast neutron radiation, and the former generally causes indel mutations in different lengths,^[^ ^61^ ^]^ while the latter often results in single base changes.^[^ ^62^, ^63^, ^64^ ^]^ Abundant mutant collections have been generated in plants such as poplar,^[^ ^61^ ^]^ *A. thaliana*,^[^ ^62^ ^]^ and rice^[^ ^63^, ^64^ ^]^ by physical radiation, providing useful resources for functional genomics. For example, using a fast‐neutron‐induced rice mutant population, Li et al. identified the *Dwarf 1/RGA1* gene as a key determinant affecting plant height and grain length.^[^ ^64^ ^]^ Chemical treatment‐based mutagenesis in plants commonly utilizes the ethyl methane sulfonate (EMS) reagent, frequently causing the Cytidine to Thymidine transition point mutation. EMS‐mediated mutagenesis has been harnessed to generate various mutant collections in plants, e.g., rice,^[^ ^65^ ^]^ maize,^[^ ^66^ ^]^ tomato,^[^ ^67^ ^]^ barley,^[^ ^68^ ^]^ and rapeseed.^[^ ^69^, ^70^ ^]^ Jiang et al. used an EMS‐induced barley mutant collection to uncover that the *HvPORB* gene was the causative factor of chlorotic trait.^[^ ^68^ ^]^ On the other hand, biological insertional mutagenesis in plants ordinarily leverages T‐DNA^[^ ^71^, ^72^, ^73^, ^74^ ^]^ or insertional boxes^[^ ^75^, ^76^ ^]^ carrying antibiotic resistance genes, transposon,^[^ ^77^, ^78^ ^]^ and retrotransposon.^[^ ^79^, ^80^, ^81^, ^82^, ^83^ ^]^ For example, Li et al. generated 62,389 insertional algal mutants, and they used the mutants to perform high‐throughput functional screening of genes required for photosynthesis, discovering that the *CPL3* gene was necessary for accumulating multiple photosynthetic protein complexes in the unicellular algal.^[^ ^75^ ^]^

Tools for targeted mutation in plants include zinc‐finger nucleases (ZFN), transcription activator‐like effector nucleases (TALEN), and clustered regularly interspaced short palindromic repeat (CRISPR)‐associated (Cas) nuclease systems, enabling the creation of mutants for elite crop breeding and gene function discovery. For example, Wang et al. utilized TALEN and CRISPR‐Cas9 to edit three homoeologs of the *TaMLO* gene simultaneously to confer heritable resistance to powdery mildew in bread wheat.^[^ ^84^, ^85^ ^]^ ZFN and TALEN generally encompass DNA recognition proteins (ZF and TALE) and DNA cleavage nucleases, e.g., FokI, to effectively function in targeted genome editing.^[^ ^86^ ^]^ Unlike the protein‐dependent DNA recognition of ZFN and TALEN, the CRISPR‐Cas system harnesses RNA molecules to recognize the target sites and one or several Cas proteins to cleave the double‐stranded DNA, enabling high‐throughput screening in plants more feasible and less challenging.^[^ ^86^, ^87^, ^88^ ^]^

### 2.4 A Comparison of the Methods Above

A comparison of the characteristics of the methods mentioned above is shown in **Table** **1**. The gain‐of‐function technique, including CRISPRa, activation tagging, and FOX‐hunting, is amenable to generating large‐scale plant transgenic collections. Subsequent identification of causative gene(s) using CRISPRa and FOX‐hunting is straightforward. However, more experimental steps for transgenics with activation tags are required to confirm the gene(s) responsible for the phenotypes, including cloning the putative locus and verifying it by overexpression, knockdown, or knockout. Sometimes, it is tricky to derive the related gene(s) because the distance of interactions between cis‐regulatory elements could be tens of mega‐bases.^[^ ^89^ ^]^ Albeit the ease of large‐scale application of the gain‐of‐function technique, the created crop transgenics do not comply with the food‐use regulatory policy.^[^ ^90^ ^]^ In addition, the exact function of the overexpressed genes could sometimes be masked due to the ectopic expression.

Except for VIGS, the knockdown strategy can also be used for the large‐scale creation of transgenic plants. Nonetheless, VIGS can be harnessed for arrayed transformation on a small scale.^[^ ^35^ ^]^ All methods for knockdown strategy own the advantage of the ease of identifying the causative gene(s), which solely requires RT‐qPCR and other simple molecular experiments. Besides, they can reduce the expression level of multiple genes based on the homology, even though lhpRNA transgenes occasionally cause off‐target silencing.^[^ ^59^ ^]^ However, the knockdown strategy also has several shortcomings. The predominant one is the incomplete knockout trait because some functional proteins are still translated. Transgenic crops created via this strategy cannot be stably inherited and do not comply with the food‐use regulatory policy.^[^ ^90^ ^]^

Conversely, crop resources produced by physicochemical treatment‐mediated random mutagenesis comply with the food‐use regulatory policy in many countries.^[^ ^90^ ^]^ Besides, leveraging physicochemical treatment to generate large‐scale plant mutant collections is relatively easy and nearly costless. However, because mutations induced by physical or chemical reagent treatment are random and numerous, identifying the causative ones requires backcrossing to obtain a mapping population, followed by massively parallel sequencing.^[^ ^68^, ^91^ ^]^ In contrast, biological insertional mutations such as T‐DNA are more effortless to identify by amplifying and sequencing the flanked sequences of known fragments. Despite the ease of using the random mutagenesis strategy, they malfunction with mutagenizing multiple homologs with redundant roles.

On the contrary, targeted mutagenesis using ZFN, TALEN, and CRISPR‐Cas systems is competent in editing various homologs. CRISPR‐Cas system, especially CRISPR‐Cas9, is quite suitable for genome‐wide screening in plants; however, it is challenging to implement TALEN and ZFN systems for large‐scale applications because they rely on proteins to recognize target sequences.^[^ ^86^ ^]^ Nonetheless, the CRISPR‐Cas system has several drawbacks. First, the potential emergence of the off‐target effect in plants will interfere with the inference of linking the edited target gene(s) to observed phenotypes. In the case of detected off‐target results, higher‐generation mutants are needed to verify the cosegregation of phenotypes and the on‐target edited gene(s) or off‐target edited gene(s). However, the off‐target effect, on the other hand, will increase the throughput in pooled CRISPR knockout screening, as shown in our previous work^[^ ^92^ ^]^ and the study in tomatoes.^[^ ^93^ ^]^ Albeit the fact mentioned above, the off‐target phenomenon is rare in plants,^[^ ^94^ ^]^ and it is predictable and detectable and can be avoided by the rational design of sgRNA.^[^ ^95^ ^]^ For example, we only identified one site with off‐target editing among the 19 ones using CRISPR‐Cas9.^[^ ^92^ ^]^ Second, chimeric mutation and genetic instability are often observed.^[^ ^92^, ^96^ ^]^ For instance, we detected 17 genes with chimeric mutation among rapeseed's 93 edited target genes, thus representing a chimera rate of 18.3%.^[^ ^92^ ^]^ Chimeric editing is putative to be a somatic mutation, and some alleles might not be transmitted to descendants. Herein, phenotypes sometimes caused by chimeric alleles are unstable across multiple generations. Besides, genetic instability also makes the association of the causative gene(s) to the phenotypes unreliable. Last but not least, the limit of the protospacer adjacent motif (PAM) and the significant variation in the editing efficiency of sgRNAs restrict the broader, easier usage of the CRISPR‐Cas system. Future studies are necessary to overcome these shortcomings.

## 3 Multi‐targeted Strategies Using the CRISPR‐Cas9

**Figure 1:**
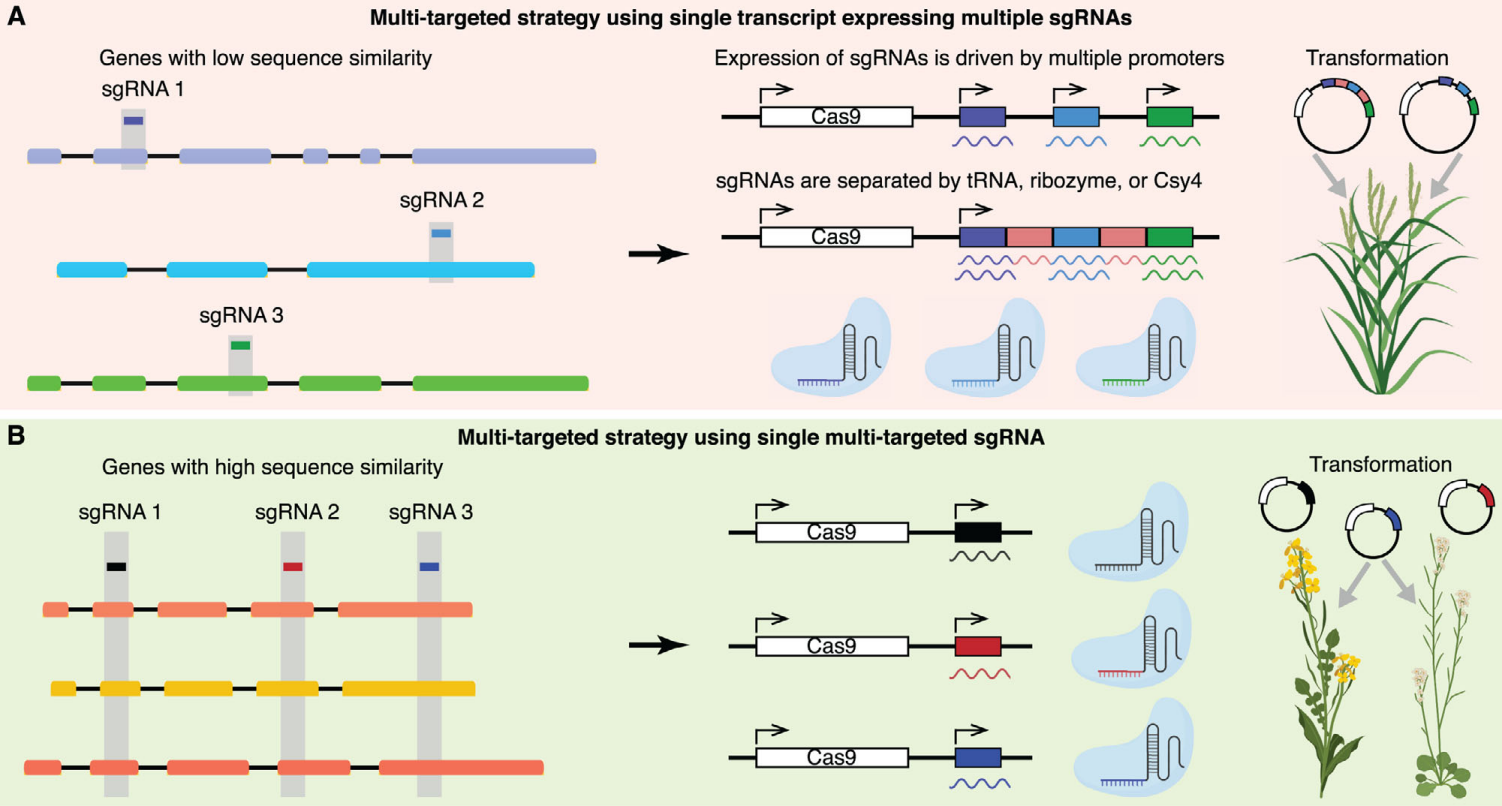
Distinct multi‐targeted strategies leveraged in plants. A) Various sgRNAs are designed to target the genes with low sequence similarity. These sgRNAs are coexpressed in the same construct driven by multiple promoters or one promoter. B) Multi‐targeted sgRNAs are designed in the conserved domains of genes with high sequence similarity. The expression of individual sgRNA per vector is driven by one promoter. The figure does not show illustrations of the promoters and other functional elements in the construct.

Polyploidization and subsequent diploidization are prevalent in flowering plants,^[^ ^97^, ^98^, ^99^, ^100^, ^101^ ^]^ typified by the massive gene families containing members with redundant functions. To leverage the multiplex genome editing advantage of the CRISPR‐Cas system, we describe the commonly used multi‐targeted strategies exemplified by CRISPR‐Cas9. These methods could be divided into two categories, i.e., single transcript expressing multiple sgRNAs and single transcript expressing a multi‐targeted sgRNA (**Figure** **1**). The two strategies could also be combined, relying on specific use cases. Besides, other characteristics acquired from previous knockout experiments in plants could also be utilized to further enlarge the number of simultaneously targeted genes.

### 3.1 Single Transcript Expressing Multiple sgRNAs

To mutagenize various genes in parallel with low sequence similarity using CRISPR‐Cas9, distinct sgRNAs particular to each gene could be simultaneously expressed in a single transcript (Figure 1A). The expression of multiple sgRNAs could be driven by their corresponding independent promoters. For instance, Xu et al. used three sgRNAs to target *GW2*, *GW5*, and *TGW6* genes for improving grain weight, which were putative to regulate grain weight negatively.^[^ ^102^ ^]^ The expression of three sgRNAs was driven by OsU3, OsU6, and TaU3 promoters, respectively. They obtained 20 T_0_ plants harboring targeted editing in all three genes among the 21 total T_0_ plants, and one line contained mutations in *GW5* and *TGW6*, thus representing an editing efficiency of almost 100%.^[^ ^102^ ^]^ The expression of multiple sgRNAs could also be driven only by a single promoter. In this case, sgRNAs are separated by tRNA,^[^ ^103^, ^104^, ^105^ ^]^ RNA endoribonuclease Csy4,^[^ ^106^, ^107^ ^]^ or self‐processing hammerhead ribozyme^[^ ^108^ ^]^ into a tandem array. After transcription, individual sgRNAs are released by endogenous processing mechanism or self‐cleavage (Figure 1A). Multisite and multigenes targeted mutagenesis could also be achieved using two tandem arrays in one vector whose expression is driven by two independent promoters. For example, Ma et al. used two sgRNA‐tRNA arrays to simultaneously target *BoMS1* and *BoSRK3* genes in cabbage to enable the economic propagation of the male‐sterile line.^[^ ^103^ ^]^ There were four sgRNAs per tandem array in their single construct. The author showed that the editing efficiency of the *BoSRK3* gene (72.2%) was higher than that of the *BoMS1* gene (27.8%) and that the simultaneous mutation efficiency of both genes was 27.8%. Small‐scale CRISPR library‐based knockout screening in plants could leverage the strategy above. However, this strategy is unsuitable for large‐ or genome‐scale screening as building large‐number constructs is burdensome.

### 3.2 Single Transcript Expressing a Multi‐targeted sgRNA

For multiple genes with high sequence similarity, the multi‐targeted sgRNA could be designed to recognize the conserved domain of these genes (Figure 1B). For example, we previously picked up three and one sgRNAs to target the conserved domains of the five and four homoeologs of the *B. napus LPAT2* and *LPAT5* genes, respectively.^[^ ^109^ ^]^ Our results revealed that all the homoeologs of the two genes were effectively knocked out by their corresponding sgRNAs (up to 41% editing efficiency of *LPAT2*, 68% editing efficiency of *LPAT5*, and no editing detected on the 14 selected putative off‐target sites), demonstrating the capacity of this strategy to edit multiple genes simultaneously solely using a multi‐targeted sgRNA.^[^ ^109^ ^]^ We also revealed that both genes commonly, but differently, facilitated seed oil accumulation in *B. napus*.^[^ ^110^ ^]^ Using a single transcript expressing one multi‐targeted sgRNA is especially useful in knocking out multiple members of gene families or homoeologs in polyploid plants. This strategy is also suitable for large‐scale CRISPR library knockout screening in plants because it is feasible to synthesize enormous sgRNAs simultaneously using the array‐based method and construct the plasmid libraries via pooled ligation.

### 3.3 Strategies to Pile Up Targeted Genes

The two methods described above have shown the solid capability for simultaneously targeting numerous genes and producing high‐order mutants in one generation.^[^ ^92^, ^111^, ^112^, ^113^ ^]^ For example, to investigate the limit of multiple irrelevant gene loci that can be targeted at once, Stuttmann et al. constructed a vector expressing nine distinct sgRNAs driven by individual *At*U6‐26 promoter to target eight genes in tobacco,^[^ ^111^ ^]^ and they got the octuple mutants at a high frequency (100% of the analyzed plants). The authors then used a construct expressing 24 sgRNAs to target 12 genes in *A. thaliana*, and they only recovered one duodecuple mutant, suggesting that Cas9 availability might restrict such higher‐order multiplexing applications.^[^ ^111^ ^]^ The authors also showed that assembling the nuclease constructs expressing up to 32 sgRNAs is feasible, thus indicating the ability to target more genes simultaneously.^[^ ^111^ ^]^ Another group used a sgRNA‐tRNA polycistron that harbored 24 sgRNAs to target 12 genes in tomato, and the authors simultaneously edited eight genes at an efficiency of 10%.^[^ ^112^ ^]^ There seems to be no position effect that affects the editing efficiency of sgRNAs on constructs in a different order, as proven by a previous study that used six sgRNAs to target six nonidentical genes.^[^ ^113^ ^]^ Our study showed that one sgRNA can simultaneously target up to nine homoeologs in *B. napus*.^[^ ^92^ ^]^ We also recovered one *fad3* mutant harboring all six mutated homoeologs mediated by a sgRNA. The results above show that we can theoretically target 288 loci at once using a construct expressing 32 different sgRNAs that each target nine homoeologs. However, the simultaneously targeted loci can be further piled up using the two tricks below. One strategy is to further enlarge the number of co‐expressed sgRNAs by transforming the recipient plants expressing the Cas9 protein so that more sgRNAs can occupy the spare space taken by Cas9, as shown in one previous study.^[^ ^114^ ^]^ The other method is to harness pooled *Agrobacterium* solution containing two distinct T‐DNA constructs in an equal amount to transform the recipient plant, as shown in our previous study.^[^ ^92^ ^]^ These strategies might help manipulate the genetic circuit of polyploid plants. However, the number of edited loci at once might be far below that of targeted loci because of many limiting factors, such as Cas9 availability. Besides, the off‐target risk will also increase due to the high similarity between the off‐target loci and the target ones.

## 4 High‐Throughput CRISPR Knockout Screenings in Plants

**Figure 2:**
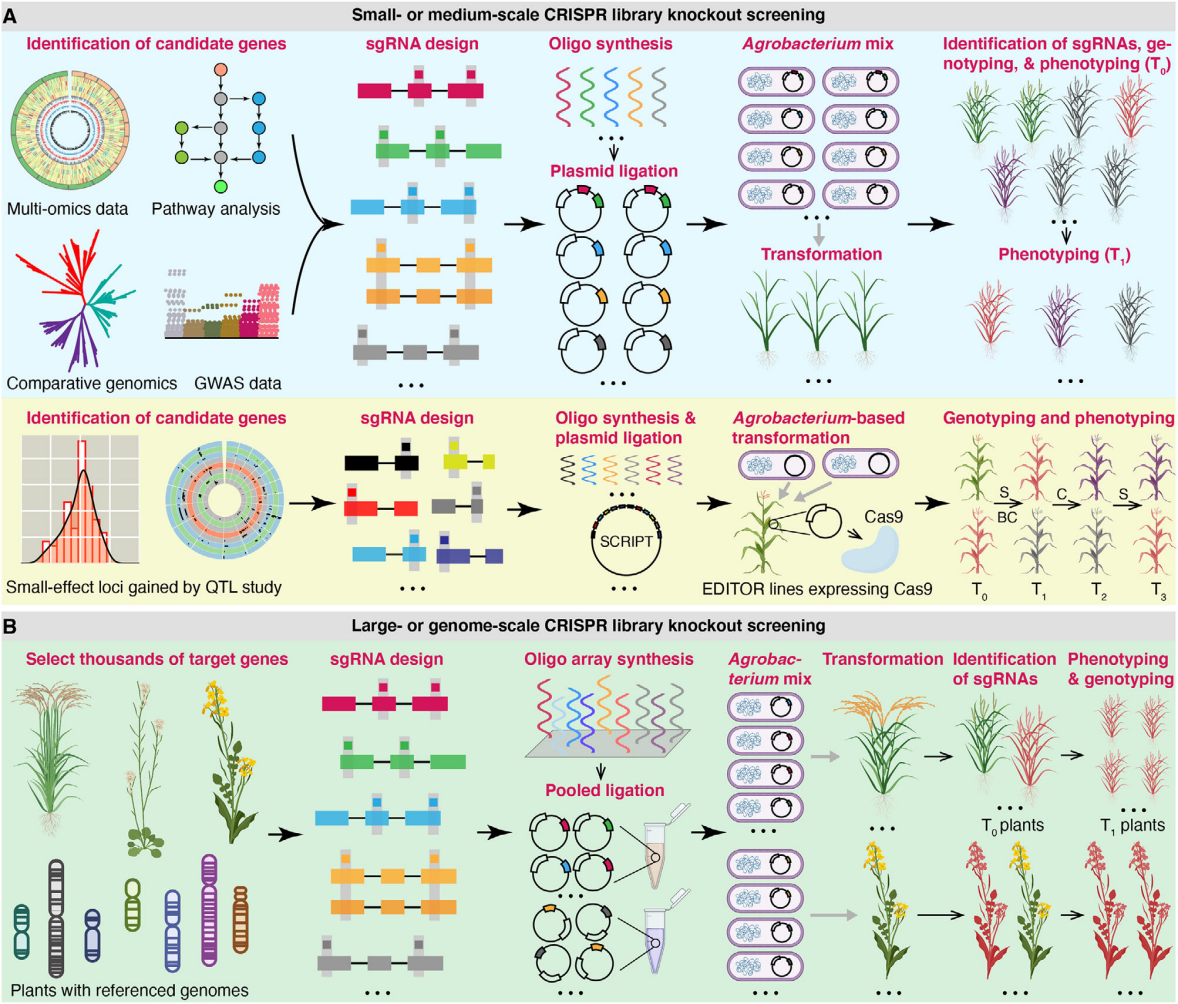
The schematic diagram displays the general pipeline for establishing plant knockout mutant collections based on CRISPR libraries. A) Small‐ or medium‐scale CRISPR library knockout screening in plants. In the lower panel, S, C, and BC are selfing, crossing, and backcrossing, respectively. B) Large‐ or genome‐scale CRISPR library knockout screening in plants. This figure does not display illustrations of the promoters and other functional elements in the plasmid.

High‐throughput CRISPR‐mediated knockout screenings in plants reported by recent studies could be divided into two categories, i.e., small‐ or medium‐scale screening and large‐ or genome‐scale screening (**Figure** **2**). This classification depends on the size of CRISPR libraries, the number of target genes, and the disparity of generating mutant collections in practice. Small‐ or medium‐scale CRISPR knockout screening in plants is frequently applied to targeted mutagenesis of tens or hundreds of genes of interest. In contrast, large‐ or genome‐scale screening is principally leveraged to mutagenize thousands of loci. Below, we comprehensively discuss the experimental differences and summarize the handful of relevant reports that leveraged CRISPR‐Cas9 to establish the knockout mutant collections (**Table** **2**).

**Table 2:** Plant knockout mutant collections generated by CRISPR libraries

Scale	Species	sgRNA library size	Target genes	sgRNAs per vector	Size of mutant collections	Refs.
Small	Tomato	165	54	1	31 (T_0_)	[93]
Small	Tomato	36	18	3	59 (T_0_)	[93]
Small	Soybean	70	102	1	407 (T_0_)	[96]
Small	Cotton	116	112	1	> 800 (T_0_)	[120]
Small	Maize	48	48	12	> 1000 (edited)	[114]
Small	Maize	191	98	2	886 (T_0_)	[121]
Medium	Maize	1186	1181	1	3470 (T_0_)	[121]
Medium	Rice	1166	1072	1	5039 (T_0_)	[122]
Medium	Tobacco	4382	1060	1	10,682 (T_0_)	[123]
Large	Rapeseed	18414	10,480	1	1104 (T_0_)	[92]
Large	Rice	88,541	34,234	1	91,004 (T_0_)	[124]
Large	Rice	25,604	12,802	1	> 14,000 (T_0_)	[125]
Large	*Arabidopsis*	59,129	16,152	1	> 3500 (T_1_)	[126]

### 4.1 Small‐ or Medium‐Scale CRISPR Library Knockout Screenings

In small‐ or medium‐scale CRISPR knockout screening, the candidate genes are often identified by diverse methods, such as multi‐omics data, metabolic pathway analysis, comparative genomics, genome‐wide association study (GWAS), and quantitative trait locus (QTL) mapping (Figure 2A).^[^ ^115^, ^116^, ^117^, ^118^, ^119^ ^]^ The size of sgRNA libraries generally ranges from dozens to one thousand, and the sgRNA oligonucleotides (oligos) are synthesized and ligated into linear constructs individually (sometimes pooled ligation is utilized). After electroporation of plasmids into competent *Agrobacterium*, cells with distinct vectors are pooled with equal amounts and then transformed into the recipient plants. Identification of sgRNAs is then carried out in the regenerated transgenic plants of the T_0_ generation. Generally, one sgRNA is represented by three or more independent T_0_ plants, enabling the association of phenotypes to the edited loci with high probability at this stage. Thus, interrogation of editing events and phenotyping could be conducted starting from the plants of the T_0_ generation (Figure 2A). For those small‐effect loci that affect the quantitative traits and are pinpointed by QTL mapping, a super vector expressing multiple sgRNAs could be used to transform the transgenic lines expressing the Cas9 to accumulate the edited alleles in one plant. The edited alleles could be further piled up in a single mutant plant by selfing, backcrossing, and crossing in successive generations (Figure 2A).

Recently, small‐scale CRISPR library knockout screening has been shown in tomato,^[^ ^93^ ^]^ soybean,^[^ ^96^ ^]^ cotton,^[^ ^120^ ^]^ and maize,^[^ ^114^ ^]^ providing valuable resources for gene function discovery and crop breeding. Jacobs et al. designed 165 sgRNAs to target 54 genes of the leucine‐rich repeat (LRR) sub‐family XII (LRR‐XII) and the control gene encoding phytoene desaturase (PDS) in tomato.^[^ ^93^ ^]^ They obtained 31 regenerated T_0_ plants and revealed an editing efficiency of 62.5%. The authors also identified off‐target editing in two lines of the T_1_ generation. Furthermore, they constructed a higher‐order library with 36 sgRNAs (three sgRNAs per vector) to target 18 genes encoding putative transporters. They regenerated 59 T_0_ plants and discovered that editing the gene of *Solyc06g071500* showed boron deficiency traits using multiple independent T_0_ plants. In soybean,^[^ ^96^ ^]^ 70 sgRNAs were designed to target 74 genes potentially involved in nodulation and 28 genes that might function in seeds (102 genes in total). Among the sgRNAs, 26 sgRNAs are highly specific, and each of the rest simultaneously targets two or more paralogous genes within their conserved regions. The sgRNA synthesis and plasmid ligation were conducted individually. The authors then divided the 70 sgRNAs into 16 pools (three to five sgRNAs were mixed in each) that were transformed into the soybean with 16 batches. Among the 407 regenerated T_0_ plants, around half contained single sgRNAs, one‐third harbored two sgRNAs, and the rest had three or more sgRNAs. Using this generated mutant collection, the authors revealed an editing efficiency of 59.2% and related the phenotype changes in nodule number to several genes while no off‐target editing was detected. Interestingly, the author used sgRNA‐specific forward primers (SSP) and a universe reverse primer to perform SSP PCR to identify sgRNAs in T_0_ plants, which differs from the report above that used Sanger sequencing. Similarly, to reveal the vital genes that might improve fertility in upland cotton, Ramadan et al.^[^ ^120^ ^]^ designed 116 sgRNAs to target the 112 genes related to the anther response toward high temperature, and the candidate genes were selected based on their transcriptome data. Total sgRNAs were divided into four sub‐libraries, and each pool contained 29 sgRNAs. The authors finally regenerated over 800 T_0_ plants, and 85% bore single sgRNA. Their editing interrogation data showed an editing efficiency of 58.9%, and deletion occupied a significant proportion of the total mutation types. In another study,^[^ ^114^ ^]^ Lorenzo et al. introduced the BREEDIT pipeline to edit the numerous small‐effect loci to accumulate the mutated alleles and improve the complex traits in maize. The authors designed 48 sgRNAs to target 48 maize growth‐related genes. The 48 sgRNAs were cloned into four SCRIPT super vectors, and each construct contained 12 sgRNAs whose expression was driven by twelve independent promoters. They first established an EDITOR line that expressed Cas9 as the recipient and finally generated over 1000 edited plants after selfing, backcrossing, and crossing to pile up the edited alleles. The authors then used this mutant collection to interrogate various traits under distinct conditions and derived a putative regulatory network.

For medium‐scale CRISPR library knockout screening, three reports have been seen in maize,^[^ ^121^ ^]^ rice,^[^ ^122^ ^]^ and tobacco.^[^ ^123^ ^]^ In maize, 1244 genes were chosen for the targeted editing, among which 98 genes were pinpointed by fine mapping, and 1181 genes were mainly from 70 QTL regions corresponding to 27 agronomically relevant traits.^[^ ^121^ ^]^ The authors then designed 1368 sgRNAs to target the 1244 candidate genes and used two strategies to pool the sgRNAs, i.e., pooling after construction and pooling after ligation. Their sequencing results showed no significant differences in sgRNA representation between the pooling methods. The authors finally regenerated 4356 T_0_ plants, revealing that 73.2% had single sgRNAs and 21.5% carried two sgRNAs. The authors also found that deletion occupied 60% of the total editing types and that mosaic mutations were widely observed in the study. Using this collection, the author showed an editing efficiency of over 80% and identified only ten loci with modification among 39,328 potential off‐target sites via whole‐exome sequencing. In rice,^[^ ^122^ ^]^ the authors put forward a FLASH pipeline to construct an arrayed CRISPR library for targeted editing 1072 receptor‐like kinase (RLK) family members. They designed 1166 sgRNAs and cloned them into 12 constructs differing in the length of the FLASH tag with 100 bp. They pooled 12 sgRNAs into one group such that sgRNAs could be identified by discriminating the size of the FLASH tag by gel electrophoresis instead of sequencing. The authors generated 5039 T_0_ plants, revealing that 96.7% had single sgRNAs and 2.4% carried two sgRNAs. Their genotyping results showed an editing efficiency of over 90% in the T_0_ generation and three off‐target editing events in the selected 37 T_0_ lines. Because many of the RLK genes were represented by three or more independent knockout lines, the collection enabled the fast discovery of defense‐related genes. Using this strategy, the authors also unraveled 2.7% of unintended editing events because of the transient expression of T‐DNA. In tobacco,^[^ ^123^ ^]^ the authors designed 4382 optimal sgRNAs to target 1060 gene locus, and then they regenerated 10,682 transgenic lines, among which 91.5% contained T‐DNA insertion. Harnessing this positive transgenic collection, the authors revealed that 57.3% carried single sgRNA and showed an editing efficiency of 52.6% evaluated with the single sgRNA‐carried lines. Finally, they attributed several phenotypes, such as muti‐branching and changes in leaf shape, to specific targeted knockout genes.

### 4.2 Large‐ or Genome‐Scale CRISPR Library Knockout Screenings

On the contrary, CRISPR libraries in large‐ or genome‐scale screening typically harbor at least ten thousand sgRNAs that target a high proportion of genes to the total ones. As a result, thousands of sgRNA oligos are synthesized simultaneously by an array‐based approach, followed by the pooled ligation of these oligos into the linear vectors and the transformation of the pooled plasmids into the competent *Agrobacterium* (Figure 2B). In this regard, one construct only carries a single sgRNA; in addition, evaluation of the coverage, correctness, and equal representation of the sgRNAs with high‐throughput or Sanger sequencing is needed for all steps before the plant transformation. A barcode‐based high‐throughput amplicon sequencing is commonly used to identify the sgRNAs in the transgenic plants of the T_0_ generation. However, obtaining a mutant collection covering each sgRNA three times or more is laborious, considering the massive amount of the CRISPR library. Therefore, the depth of the sgRNA coverage by the independent T_0_ plants is usually 1X. In this regard, it is risky to attribute the observed phenotypes to the edited genes. Herein, we recommend conducting phenotyping and genotyping using at least ten T_1_ plants in a high‐throughput manner to link the phenotypes to the causative mutated genes robustly (Figure 2B).

Only four large‐ or genome‐scale CRISPR libraries were seen in rice,^[^ ^124^, ^125^ ^]^ rapeseed,^[^ ^92^ ^]^ and *Arabidopsis*.^[^ ^126^ ^]^ In rice, two studies in parallel reported the genome‐wide targeted mutagenesis using the large‐scale CRISPR library. Lu et al. designed 88,541 sgRNAs to target 34,234 protein‐coding genes, which occupied 83% of the rice genome.^[^ ^124^ ^]^ The authors generated 91,004 T_0_ plants and used barcode‐based high‐throughput amplicon sequencing to identify the sgRNAs. The size of the transgenics collection corresponded to 1X sgRNA coverage. The authors also revealed an editing efficiency of around 80% and discovered several mutants with abnormal phenotypes. The other research group used 25,604 sgRNAs to target the 12,802 highly expressed genes in shoot tissues, and they obtained over 14,000 T_0_ plants, corresponding to 0.5X sgRNA coverage.^[^ ^125^ ^]^ Using the transgenics collection, the authors showed an editing efficiency of 63.3% and no mutation in the 22 predicted off‐target sites. Our group successfully demonstrated the feasibility of leveraging a large‐scale CRISPR library to achieve targeted mutagenesis in the allotetraploid crop of *B. napus*.^[^ ^92^ ^]^ We designed 18,414 sgRNAs to target the 10,480 genes related to the development of the reproductive organ. One sgRNA could simultaneously target up to nine homoeologs. We generated a transgenic collection of 1104 T_0_ plants and used barcode‐based high‐throughput amplicon sequencing to identify the sgRNAs. We then revealed that 88.3% of the T_0_ plants contained single sgRNAs, and 11.1% harbored two or three sgRNAs. We next showed an editing efficiency of around 50% of this collection and observed mosaic mutations in 17 genes. Interestingly, we also discovered that Cas9‐mediated DNA double‐stranded breaks tend to occur at all the target sites guided by the single sgRNA. Finally, we used forward and reverse genetics studies to interrogate this mutant collection and reveal several novel genes that might dominate seeds' oil content and fatty acid composition. To overcome the gene functional redundancy in *A*. *thaliana*, another research group designed 59,129 optimal sgRNAs to target 16,152 genes that belong to families.^[^ ^126^ ^]^ One sgRNA could simultaneously target two to ten gene‐family members. The authors then partitioned the library into ten sub‐libraries according to the protein functions of the putative target genes. They used the pooled CRISPR library consisting of 5635 sgRNAs targeting the plant transportome to generate over 3500 independent *Arabidopsis* lines. The authors finally characterized the redundant functions of three novel genes, i.e., *PUP7*, *PUP8*, and *PUP21*.

## 5 Future Perspective

Different scales of high‐throughput CRISPR library knockout screenings are booming in plant biology research. The aforementioned reported studies have displayed distinct characteristics in mutation efficiency, percentage of chimeric mutations, the number of sgRNAs per plant, the edited types, and so on in divergent plants. As a result, several critical factors should be considered when leveraging the CRISPR library for gene knockout screening.^[^ ^127^ ^]^ In our experience of creating a rapeseed mutant collection by a pooled CRISPR library, the editing efficiency of CRISPR‐Cas9 and the regeneration efficiency of transgenics by tissue culture are two vital limited factors.^[^ ^92^, ^128^ ^]^ Optimizing the CRISPR‐Cas9 system to improve its editing efficiency in plants,^[^ ^129^ ^]^ such as machine learning‐facilitated editing outcome prediction and rational sgRNA design,^[^ ^127^ ^]^ will be required in future studies. In addition, novel plasmid delivery methods bypassing tissue culture, such as virus‐based^[^ ^130^, ^131^ ^]^ and carbon nanocarrier‐based delivery^[^ ^132^, ^133^, ^134^ ^]^ and de novo induction of meristems,^[^ ^135^ ^]^ might be used in arrayed CRISPR library screening in the future. Alternatively, improving the regeneration efficiency of transgenic plants is also beneficial to high‐throughput pooled CRISPR library knockout screenings.^[^ ^136^, ^137^, ^138^, ^139^ ^]^ For instance, overexpressing only *TaWOX5* can improve the transformation efficiency in wheat, einkorn, triticale, rye, barley, and maize.^[^ ^139^ ^]^

In addition to exploring and utilizing new CRISPR‐Cas systems, optimizing the most frequently used CRISPR‐Cas9 system to overcome its shortcomings is vital for various applications, especially for plant functional genomics. First, Cas9 variants created by protein engineering are necessary to relax the PAM constraint of NGG. For instance, a near‐PAMless Cas9 variant denoted as SpRY (NRN and, to a lesser extent, NYN PAMs) was created^[^ ^140^ ^]^ and successfully used in rice and Dahurian larch.^[^ ^141^ ^]^ Second, unearthing the Cas9 variants with improved editing efficiency and reduced off‐target activity will benefit large‐scale CRISPR knockout screening in plants. For instance, Choi et al.^[^ ^142^ ^]^ used combinatorial mutagenesis to assess the on‐ and off‐target editing at multiple sites of a pool of SpCas9 variants. They identified a Cas9 mutant, Opti‐SpCas9, with reduced off‐target potency (1.7% of WT activity) without sacrificing on‐target editing efficiency (94.6% of WT activity). The optimized CRISPR‐Cas9 systems, beyond knockout or knockin, will show a broad range of plant applications in future studies. For instance, it can be leveraged to build endogenous gene connections by employing a trigger RNA strand, such as small RNAs, to induce the formation of a ternary guide RNA assembly for functional control of CRISPR‐Cas9 to manipulate the genetic circuit,^[^ ^143^ ^]^ which has yet to be reported in plants. This manipulation can be performed in specific plant cell types, tissues, or organs using the adapted CRISPR‐TSKO tool tested in *A. thaliana*.^[^ ^144^ ^]^

**Figure 3:**
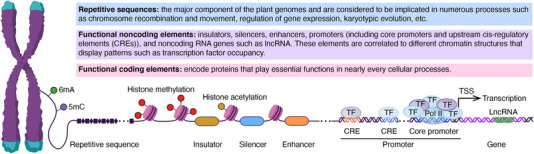
The picture illustrates the repetitive sequences and functional elements in the plant genomes. (TF) transcription factor, (TSS) transcription start site, (LncRNA) long noncoding RNA, and (Pol II) RNA polymerase II.

Aside from functional coding elements encoding proteins, the roles of most functional noncoding components and repetitive sequences remain uncharacterized (**Figure** **3**). Functional noncoding elements include insulators, silencers, enhancers, promoters, and noncoding RNA genes, such as long noncoding RNAs (lncRNAs), related to different chromatin structures that display patterns such as transcription factor occupancy.^[^ ^145^ ^]^ Only a few studies have been reported to manipulate these plant components via genetic engineering to decipher their roles in regulatory networks.^[^ ^146^, ^147^, ^148^, ^149^, ^150^, ^151^ ^]^ For example, Liu et al. used CRISPR‐Cas9 to edit the promoter of *CLE* genes to enhance grain‐yield‐related traits in maize.^[^ ^146^ ^]^ For lncRNAs that are considered to play vital roles in plant immunity and diverse plant‐microbe or insect interaction,^[^ ^152^ ^]^ two lncRNAs were mutated with the CRISPR‐Cas9 system, leading to a negative effect on aphid resistance and a decrease of jasmonic acid content.^[^ ^151^ ^]^ However, high‐throughput CRISPR library interrogation of these elements in plants has yet to be reported, and only one such study was found to be carried out in *Bombyx mori* targeting promoters with a large‐scale sgRNA pool.^[^ ^153^ ^]^ Repetitive sequences, the major component of the plant genomes,^[^ ^154^ ^]^ also remain largely uncharacterized. They are implicated in a wide variety of biological processes, such as chromosome recombination and movement, gene expression regulation, and karyotypic evolution.^[^ ^155^ ^]^ Recently, a study reported using a large‐scale CRISPR library comprising 566,766 gRNAs to deplete the repeats in *Lens culinaris* for high‐throughput genotyping of complex plant genomes.^[^ ^156^ ^]^ Choosing a suitable CRISPR‐Cas tool from the toolbox^[^ ^157^ ^]^ for high‐throughput CRISPR library interrogation of functional coding or noncoding elements and repetitive sequences will be prevalent in future studies. For instance, the fact that CRISPR‐Cas12j2‐mediated editing frequently induces roughly 9‐bp deletions makes it useful in promoter editing.^[^ ^158^, ^159^ ^]^ The compact Cascade‐Cas3 Dvu I‐C system with Cas11c can efficiently introduce controllable large fragment deletion up to at least 20 kb, making it useful for exploring the function of regulatory elements.^[^ ^160^ ^]^ We believe these mutant collections to be built will accelerate plant functional genomics and crop breeding in the future.

## Conflict of Interest

The authors declare no conflict of interest.

## Author Contributions

J.H. performed the analyses, prepared the figures and table, and drafted the manuscript. C.Z. retrieved the data and conducted part of the analyses. M.L. supervised the study and acquired funding.
